# Airborne metals and malignant pleural effusions: the role of oxidative stress

**DOI:** 10.3389/ftox.2026.1835591

**Published:** 2026-05-21

**Authors:** Jesús Valencia-Cervantes, Gustavo Ramirez-Martínez, Margarita Isabel Palacios-Arreola, Alejandra Loaeza-Román, Martha Patricia Sierra-Vargas

**Affiliations:** 1 Departamento de Investigación en Toxicología y Medicina Ambiental, Instituto Nacional de Enfermedades Respiratorias Ismael Cosío Villegas (INER), Mexico City, Mexico; 2 Estancias Posdoctorales por México 2022 (1), Secretaría de Ciencia, Humanidades, Tecnología e Innovación (SECIHTI), Mexico City, Mexico; 3 Laboratorio de Inmunobiología y Genética, Instituto Nacional de Enfermedades Respiratorias Ismael Cosío Villegas (INER), Mexico City, Mexico

**Keywords:** air pollution, airborne metals, antioxidant system, lung cancer, malignant pleural effusion, oxidative stress, particulate matter, reactive oxygen species

## Abstract

An excess of pleural fluid production can result in a condition known as pleural effusion, characterized by the accumulation of fluid in the space between the lungs and the chest wall. Malignant pleural effusions are defined as the presence of neoplastic cells in pleural fluid. The presence of malignant pleural effusions has been attributed to lung cancer, which is present in 15% of patients at the time of diagnosis. The development of pleural effusion has been strongly linked to environmental factors, including exposure to air pollution, chemical compounds, and occupational hazards. The present study examines the association between the induction of oxidative stress in malignant pleural effusion and the presence of air pollution metals or metalloids. A search was conducted using the following databases: PubMed, Science Direct, and the Cochrane Library, to identify all original scientific articles published through 30 January 2026. The following search combination terms were utilized: trace elements, heavy metals, metalloids, particulate matter, air pollution, pleural effusion, oxidative stress, antioxidant enzymes, and lung cancer. The findings revealed a higher concentration of zinc in malignant pleural effusions compared to benign pleural effusions, a statistically significant difference. Furthermore, an elevated concentration of trace elements, including copper, chromium, lead, selenium, and iron, has been documented in malignant pleural effusions. However, these observations did not demonstrate statistical significance. With respect to antioxidant activity, an increase in superoxide dismutase activity was observed in malignant pleural effusion, as well as in the oxidative stress markers Lactate Dehydrogenase, Malondialdehyde, and Reactive Oxygen Species Modulator 1. The presence of metals or metalloids of anthropogenic origin identified in pleural effusions has the potential to increase the production of reactive oxygen species, increasing oxidative stress damage and supporting the progression of respiratory diseases, such as lung cancer.

## 1 Introduction

The pleural space contains a minimal amount of fluid, approximately 0.3 mL/kg of body weight. The predominant site of fluid accumulation is the parietal region of the pleura, with a propensity for the upper chest. Subsequently, the fluid is reabsorbed into the body through lymphatic vessels located in the lower regions of the pleural space, particularly near the diaphragm and around the heart. An excess of fluid production can result in a condition known as pleural effusion (PE), characterized by the accumulation of fluid in the space between the lungs and the chest wall (Miserocchi, 1997; Krishna et al., 2024). In accordance with Light’s criteria, the classification of PE, as either an exudate or transudate, is determined by the biochemical properties. A disruption of the local inflammatory factors that trigger a pleural fluid accumulation is defined as an exudative effusion. This phenomenon occurs when the filtration rate exceeds the maximum lymph flow capacity, resulting in an effusion with a higher-than-usual protein content. An effusion with an elevated protein content is also formed when there is an increase in protein permeability of the systemic capillaries. The etiology of these effusions is typically attributed to underlying infections, which may include, but are not limited to, pneumonia, malignancy, granulomatous diseases such as tuberculosis, collagen vascular diseases, and other inflammatory states. If filtration exceeds the maximum lymph reabsorption capacity of the parietal stomata, transudate effusion is designed. Subsequently, the fluid is propelled through the capillaries due to the elevated pressure within them. An imbalance between the hydrostatic and oncotic pressure within the capillaries can result in a transudate effusion. Moreover, a variety of clinical conditions have been observed to be associated with transudate PE, including congestive heart failure (CHF), cirrhosis, nephrotic syndrome, and malnutrition (Porcel, 2006; Kopcinovic and Culej, 2014; Bashour et al., 2022). In patients diagnosed with cancer, the presence of a PE is often indicative of a poor prognosis. The mean survival time for patients with hematologic malignancies or pleural mesothelioma is approximately 1 year, while patients with lung cancer (LC) have the poorest prognosis, with an average survival time of only two to 3 months (Jany and Welte, 2019). Malignant pleural effusions (MPE) are defined as the presence of neoplastic cells in pleural fluid. Approximately 35% of MPE are secondary to LC, with adenocarcinoma (ADE) being the most prevalent histological subcategory of LC. In addition, breast cancer is the most prevalent secondary diagnosis, present in 23% of MPE cases, while lymphomas account for up to 10% of MPE cases (Pardessus-Otero et al., 2024). According to the Global Cancer Observatory (Bray et al., 2018) platform, 2,094,000 new cases of LC were identified as the leading cause of worldwide cancer deaths, accounting for approximately 1,761,000 deaths (18.4% of all cancer-related deaths). The number of deaths attributable to LC designates this condition as the primary cause of mortality among the population in 93 countries (Thandra et al., 2021).

Air pollution is a complex mixture of substances, including particulate matter (PM). The chemical composition of PM includes trace metals and metalloids, which have been demonstrated to increase the risk of cancer, respiratory diseases, and arteriosclerosis with long-term exposure to high levels of such particles (Lodovici and Bigagli, 2011). Conversely, short-term exposure to these pollutants has been demonstrated to exacerbate bronchitis, asthma, and other respiratory diseases, as well as to induce changes in heart-rate variability. The presence of transition metals has been demonstrated to generate oxidative stress (OxS), which is a fundamental molecular mechanism underlying metal-induced toxicity. The transition metals have the capacity to undergo redox cycling reactions, which in turn can result in the production of reactive oxygen species (ROS). The excess of intracellular ROS has been demonstrated to disrupt the intracellular redox state and energy production, resulting in subsequent modifications of cellular biomolecules, including DNA, lipids, and proteins, and the dysfunction of mitochondrial respiration, protein folding, DNA repair processes, endoplasmic reticulum stress, inflammation, autophagy, and apoptosis (Chen et al., 2018).

The present study examines the association between the induction of OxS in MPE and the presence of air pollution metals or metalloids. A comprehensive literature review was conducted using PubMed, Science Direct, and the Cochrane Library to identify all original scientific articles published through 30 January 2026. The following search combination terms were utilized: “trace elements”, “heavy metals”, “metalloids”, “particulate matter”, “air pollution”, “pleural effusion”, “oxidative stress”, “antioxidant enzymes”, and “lung cancer”. The inclusion criteria were delineated as follows: studies conducted worldwide that focused on metals and metalloids present in human MPE and BPE, in conjunction with evaluations of human PE associated with LC (ADE, squamous cell carcinoma of the lung, and lung mesothelioma). The exclusion criteria were defined as follows: articles not written in English, research classified as clinical trials or meta-analyses; studies involving animal subjects; inquiries pertaining to any infectious or viral diagnostics unrelated to LC; and investigations into other types of cancer apart from LC. Although the establishment of clearly defined eligibility criteria aimed at enhancing transparency and reproducibility was evident, a formal, structured assessment of methodological quality and risk of bias of the included studies was not conducted. The absence of such an evaluation signifies a limitation of this narrative review. The presence of heterogeneity can be attributed to variations in study design, sample handling, analytical techniques, and reporting procedures. The findings revealed a higher concentration of zinc in MPE compared to benign pleural effusions (BPE), a statistically significant difference. Furthermore, an elevated concentration of trace elements, including copper, chromium, lead, selenium, and iron, has been documented in MPE. However, these observations did not demonstrate statistical significance. With respect to antioxidant activity, an increase in Superoxide Dismutase activity was observed in MPE, as well as in the oxidative stress markers Lactate Dehydrogenase, Malondialdehyde, and Reactive Oxygen Species Modulator 1. The presence of metals or metalloid of anthropogenic origin identified in pleural effusions has the potential to increase the production of ROS, increasing OxS damage and supporting the progression of respiratory diseases, such as lung cancer.

## 2 Association of air pollution with LC

Air pollution is defined as a mixture of hazardous substances originating from both anthropogenic and natural sources. The classification of sources is divided into three primary categories: Firstly, the principal source of these emissions is direct emissions from an air pollution generator. Secondly, the source materializes as a consequence of the chemical reaction of precursors emanating from air pollution sites, leading to the formation of a pollutant in the atmosphere. Thirdly, the re-emission process is initiated by the deposition of primary or secondary pollutants on the terrestrial or aquatic surfaces of the Earth (IARCWorking Group on the Evaluation of Carcinogenic Risks to Humans, 2016). The presence of various types of PM in the atmosphere, including coarse PM (PM_10_, with an aerodynamic diameter of <10 µm), fine PM (PM_2.5_, with an aerodynamic diameter of <2.5 µm), and ultrafine PM (PM_0.1_, with an aerodynamic diameter of <0.1 µm), is predominantly produced by combustion processes. These PM types have been associated with respiratory diseases, including LC, chronic obstructive pulmonary disease (COPD), and pneumonia, as well as with stroke and ischemic heart disease, among many other diseases (Mühlfeld et al., 2008). The combined effects of ambient and household air pollution have been associated with approximately 6.7 million premature deaths annually (World Health Organization, 2018).

## 3 Contribution of airborne metals and metalloids to the composition of PM_2.5_

**FIGURE 1:**
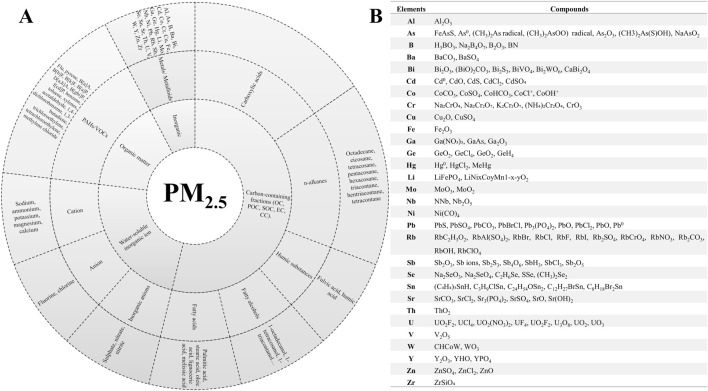
Identification of the chemical composition of PM_2.5_. **(A)** Carbon compound: organic carbon (OC), primary organic carbon (POC), secondary organic carbon (SOC), elemental carbon (EC), carbonate carbon (CC), polycyclic aromatic hydrocarbons (PAHs), volatile organic compounds (VOCs), fluoranthene (Flu), pyrene, benzo[a]anthracene (B[a]A), benzo[b]fluoranthene (B[b]F), benzo[k]fluoranthene (B[k]F), benzo[a]pyrene (B[a]P), dibenz[a,h]anthracene (D[a,h]A), benzo[ghi]perylene (B[ghi]P), indenol[1,2,3-cd]pyrene (I[cd]P. **(B)** The principal metal compounds that have the potential to induce respiratory toxicity through occupational or environmental exposure. Adapted of Lyu et al. (2019); Wu et al. (2021); Hasegawa (2022); Alves et al. (2023).

PM_2.5_ is a heterogeneous mixture of complex components produced from multiple sources. The PM_2.5_ components are comprised of water-soluble inorganic ions (account for 30%–50% of the total mass), carbon compounds as organic carbon (OC), elemental carbon (EC), and carbonate carbon (CC) (contribute 20%–50%). Among these carbon components, OC and EC account for 10%–70% of the carbon mass, while CC contributes a comparatively negligible percentage. Based on their solubility in water, the organic composition of PM_2.5_ can be categorized into two distinct groups. The first group encompasses polycyclic aromatic hydrocarbons (PAHs) and polycyclic aromatic ketones, which exhibit limited solubility in water. The second group consists of alcohols, carboxylic acids, phenols, keto acids, and hydroxylamines, which are water-soluble. These substances are classified, as persistent organic pollutants, chemicals that persist in the environment, are toxic to humans, and have the capacity to travel great distances (Lyu et al., 2019; Wu et al., 2021; Hasegawa, 2022; Alves et al., 2023). Heavy metals (HM) and metalloids exhibit a high degree of adsorption onto the PM_2.5_ surface due to their small particle size and high surface area (Jaishankar et al., 2014; Jomova et al., 2025). The most predominant elements in PM_2.5_ which include but are not limited to: Aluminum (Al), Arsenic (As), Boron (B), Barium (Ba), Bismuth (Bi), Cadmium (Cd), Cobalt (Co), Chromium (Cr), Copper (Cu), Iron (Fe), Gallium (Ga), Germanium (Ge), Manganese (Mn), Magnesium (Mg), Mercury (Hg), Lithium (Li), Molybdenum (Mo), Niobium (Nb), Nickel (Ni), Lead (Pb), Rubidium (Rb), Antimony (Sb), Selenium (Se), Silver (Ag), Tin (Sn), Strontium (Sr), Thorium (Th), Uranium (U), Vanadium (V), Tungsten (W), Yttrium (Y), and Zinc (Zn) (Jaishankar et al., 2014; Li et al., 2023; Jomova et al., 2025). HM are components and cofactors of enzymes and function in redox reactions, thereby playing pivotal roles in biochemical processes. The production of ROS and OxS has been identified as a crucial factor in toxicity and carcinogenicity. It has been demonstrated that HM can lead to toxic accumulation by forming stable and difficult-to-degrade compounds (Matei et al., 2025). As demonstrated in Figure 1A, the chemical composition of PM_2.5_ can be categorized into distinct groups, including inorganic and organic compounds, water-soluble inorganic ions, and carbon-containing fractions. It is also indicated that the presence of metal compounds has been documented to cause respiratory toxicity through occupational or environmental exposure (Figure 1B).

## 4 Airborne metals and metalloids are associated with lung diseases

The epidemiological studies have established a correlation between exposure to air pollution and mortality, with a primary focus on cardiovascular and respiratory diseases (RD). According to Lu et al. (2025), findings from 1,587 cases of RD-related fatalities indicated a notable correlation between elevated RD mortality rates and increased concentrations of PM_10_, PM_2.5_, and CO. Specifically, an increase of 10 μg/m^3^ in these pollutants was associated with a 0.7%, 1.3%, and 0.2% increase in excess mortality, respectively. The highest positive correlations between NO_2_ and O_3_ with delays of one and 2 days were observed, resulting in excess mortality risks of 2.0% and 0.9%, respectively. Similarly, Kebe et al. (2025) revealed that short-term exposure to PM_10_ was associated with a 1.2% increase in the risk of all-cause mortality across all age groups included; with a relative risk (RR) for respiratory mortality in children, under five was 1.4%. Furthermore, the assessment of HM exposure through three routes (inhalation, ingestion, and dermal contact) enabled the determination of the hazard index (HI) and carcinogenic risk (CR). In both cases, the HI values for Cr, Cu, Mn, Ni, V, and Zn were found to be below the threshold of 1. The CR values for Cr, Ni, and Pb were determined to be outside the regulatory range of 10^–6^ and 10^–4^. Furthermore, Cha et al. (2025) analyzed the spatiotemporal trends and health risks of nine atmospheric HM (Pb, As, Mn, Ni, Cr, Cd, Zn, Cu, Fe) in PM_2.5_ across 50 cities (China); the combined non-carcinogenic hazard index (HI < 1) was unable to attain safety thresholds. However, the combined carcinogenic risk total for children surpassed 10^–4^, predominantly attributable to As and Cr.

Several studies have evaluated the individual effect of metals; however, it is important to evaluate the mixture of metals, which would be a better approximation of reality, as determined by Zhang et al. (2025), the exposure to mixtures of Pb, Cd, and Hg has been demonstrated to be associated with an increased risk of all-cause cancer, cardiovascular disease (CVD), stroke, and respiratory disease mortality, with an odds ratio (OR) ranging from 1.0–1.1. Among the specific etiologies of mortality, Pb and Cd exhibited correlation with respiratory diseases (e.g., ischemic heart disease, CVD, and cancer). Gouveia et al. (2024) established that a positive correlation between a 10 μg/m^3^ increase in monthly PM_2.5_ and a concomitant rise of 1.3% in cardiovascular mortality and 0.9% in respiratory mortality was observed in Latin Americans. Similarly, Ho et al. (2019) conducted a study in Taiwan, including 30 non-COPD patients with pneumonia and 27 COPD patients with pneumonia, which showed that increases in PM_2.5_ and NO_2_ lead to an elevated risk of pneumonia in patients with COPD, with an OR of 4.1 in comparison to 1.8 in non-COPD patients. Moreover, the PE of COPD patients exhibited an increase in Cu and a decrease in Zn, which, in turn, increased the risk of pneumonia. Finally, Zhang F. et al. (2020) examined a total of 16,483 new cases of LC who resided in 36 cities (China). The RR was determined to be 1.1 for LC in relation to a 10 μg/m^3^ increase in 3-year PM_2.5_ exposure. The population attributable risk was estimated for a reduction in PM_2.5_ concentration to 35 μg/m^3^, which corresponded to a 14% decrease in cases of LC.

A variety of studies has demonstrated a correlation between the presence of metals in air pollution and their capacity to trigger various respiratory diseases, resulting in increased morbidity and mortality. The characterization of metal compounds in PM has been attributed to their ability to modulate or modify signaling pathways (e.g., ROS/OxS) that favor the progression of respiratory diseases. Therefore, it is necessary to consider the etiological impact of these metals on diseases such as LC. Table 1 presents a comprehensive list of metals associated with lung pathogenesis disorders, as well as the identification of carcinogenic risks for humans according to the International Agency for Research on Cancer (IARC).

**TABLE 1:** Metals associated with toxicity and lung pathogenesis disorders.

Metals	Mechanisms toxicity	Lung pathogenesis disorders
Al IARC: group 1 (Jomova et al., 2025; Bonfiglio et al., 2023; Renke et al., 2023)	• Disrupts or inhibits enzymatic activity, alters protein synthesis and nucleic acid function, modifies cell membrane permeability, inhibits DNA repair, degrades the stability of DNA organization, and inhibits protein phosphatase 2A activity, ROS production, and OxS damage, disrupts cellular iron homeostasis, and induces apoptosis	• Asthma, chronic bronchitis, chronic pneumonia, pulmonary alveolitis, COPD, pulmonary fibrosis, alveolar proteinases, pneumoconiosis, pulmonary granulomatosis, potroom asthma, mild-to-moderate bronchial hyperresponsiveness: wheezing, shortness of breath, dyspnea, cough, and phlegm
Cd IARC: group 1 (Jomova et al., 2025; Charkiewicz et al., 2023; Yang et al., 2025)	• ROS production and OxS damage, gene regulation, induce apoptosis, and autophagy	• Irritation of the mucous membranes of the nose: disrupting the sense of smell, pulmonary edema and chronic bronchitis, dry cough followed by expectoration, dyspnea, and symptoms resembling flu: fever, body pain
As IARC: group 1 (Jomova et al., 2025; Hong et al., 2014; Kuivenhoven and Mason, 2023)	• ROS production and OxS damage, inhibit mitochondrial respiration and uncouple mitochondrial oxidative phosphorylation, regulate glycolysis, deplete ATP, dysregulate and inhibit proteins/enzymes	• Irritation of the nasal mucosa, larynx, bronchi, and later perforation of the nasal septum, rhino-pharyngolaryngitis, trachea bronchitis, pulmonary insufficiency, chronic cough, bronchopulmonary disease, arsenic bronchitis, asthma, dyspnea, chest pain, non-cardiogenic pulmonary edema, increased capillary permeability, and LC.
Hg IARC: group 3 (Jomova et al., 2025; Park and Zheng, 2012; Charkiewicz et al., 2025)	• ROS production and OxS damage, calcium homeostasis, damage neurotransmitters, and induce neurotoxicity	• Inflammatory reaction in the lungs or airways, reduced lung function and difficulty breathing, pneumonia, bronchitis, pulmonary fibrosis, chest pain, angina, asthma, and Young’s syndrome
Pb IARC: group 2A, 2B, 3 (Jomova et al., 2025; Gonzalez-Villalva et al., 2025)	• ROS production and OxS damage, increase the levels of MDA and glutathione disulfide, decrease the antioxidant system (CAT, GPx, GR, GSH, SOD, GST), alter calcium metabolism, autophagy, and apoptosis, promote inflammation, increase alkaline phosphatase levels, decrease bone mineral density, inhibit vitamin D activation, and modify cell proliferation and cell adhesion	• Asthma and allergies, reduction in forced expiratory volume in 1 s, alterations in non-ciliated bronchiolar cells, thickening of the alveolar septa, hemorrhage, thrombosis, inflammatory cells, atelectasis, bronchiolitis, hyperemia
Cr IARC: group 1, 3 (Jomova et al., 2025; Shin et al., 2023)	• ROS production, and OxS damage, DNA damage, metastasis, cell cycle alteration, immune system disorder, alteration of ribosome biogenesis and rRNA metabolic process, modulation of cell–substrate adhesion	• Increased risk of LC, nasal irritation, pneumonia, pulmonary fibrosis, and emphysema
Fe IARC: group 1 (Haidar et al., 2023; Papanikolaou and Pantopoulos, 2005)	• ROS production/OxS damage, disrupts oxidative phosphorylation, increases lipid peroxidation, damages cellular oxidizing and reducing mechanisms, attacks DNA, resulting in cellular damage, mutation, and malignant transformations	• COPD, LC, cystic fibrosis, idiopathic pulmonary fibrosis, and asthma
Ni IARC: group 1 (Haidar et al., 2023; Zambelli et al., 2016; Genchi et al., 2020; Gates et al., 2023)	• ROS production/OxS damage, regulates pathways associated with carcinogenesis (PI3K/AKT, NF-κB, ERK, AMPK, HIF-1α), regulates glucose metabolism-associated pathways (gluconeogenesis and glycogenolysis), neurotoxicity-associated pathways (Caspase-9 by BAX, BID, and cyt c, caspase-8 and caspase-10 by Fas-FasL)	• Asthma, bronchitis, and inflammation, LC, cancers of the nose and nasal sinuses, instant symptoms: nausea, vomiting, vertigo, and irritation, followed by delayed type symptoms like stiffness of the chest, constant cough, palpitations, sweating, tachycardia, visual disturbances, weakness, rhinitis, polyps, and fibrosis
V IARC: group 2B (Haidar et al., 2023; Toxicological Profile for Toxicological Profile for Vanadium, 2012)	• Inhibitor of the Na + K + -ATPase, stimulates tyrosine kinase phosphorylase, NADPH oxidase, and adenylate cyclase, stimulates glucose uptake, oxidation, and glycogen synthesis, induces cell proliferation, increases mRNA levels encoding pro-fibrogenic growth factors (e.g., TGF-β1, CTGF, and PDGF-C) and chemokines (e.g., IFN-α, IFN-β, CXCL9, and CXCL10), increases collagen mRNA levels, increases apoptosis, with minimal lung cell necrosis	• Cough, wheezing, chest pain, runny nose, or sore throat, increased neutrophils in the nasal mucosa, signs of respiratory distress, impaired lung function, increased pulmonary reactivity, and histological alterations in the lungs, larynx, and nasal cavity, audible wheezing and coughing, fibrosis, bronchiolar epithelial hyperplasia, inflammation, damage to the larynx, degeneration and hyperplasia of the epiglottis epithelium, and squamous metaplasia of the epiglottis epithelium
Li IARC: group 2B (Hedya et al., 2023; Gbondo et al., 2025; Sakrajda and Rybakowski, 2025)	• Induces changes in Na transport, influences the metabolism of neurotransmitters (catecholamines and serotonin), diminishes the activity of protein kinase C, elevates cytoprotective proteins, and potentially triggers neurogenesis, increasing gray matter volume, affects the purinergic system, electrolyte metabolism, membrane transport, and second messenger systems	• Respiratory illness and airway obstruction
Sb IARC: group 2A, 3 (NCBI, 2019; Boreiko and Rossman, 2020; Bolan et al., 2022; Martin and Lee, 2024)	• ROS production/OxS damage, alters gene expression, and inhibitors of DNA repair, could result in persistence or error-prone repair of promutagenic lesions	• Irritation of the epiglottis epithelium, lung interstitial fibrosis, local respiratory tract irritation can lead to pneumonitis, tracheitis, and laryngitis, cases of acute respiratory distress syndrome, chronic exposure: Sb-pneumoconiosis, which may progress to COPD, soreness and bleeding of the nose, rhinitis, and laryngitis, chronic coughing, inflammation, and chronic bronchitis
Co IARC: group 2A, 2B (Catalani et al., 2012; Leyssens et al., 2017; NCBI, 2024; Chen and Lee, 2026)	• ROS production/OxS damage, inhibits DNA repair and genotoxicity, blocks the inorganic Ca channels, interferes with normal homeostatic degradation of HIF-1α; exposure to Co can often mimic hypoxic conditions and increases lipid peroxidation	• Pneumoconiosis, ‘hard metal disease’ (hard metal asthma), occupational asthma, allergic alveolitis, hypersensitivity pneumonitis, and interstitial pneumonia, weight loss, fatigue, dry cough, dyspnea on exertion, chest pain, wheezing, and rales, cyanosis, digital clubbing, pulmonary hypertension, signs of right heart failure, and pulmonary dysfunction can be of the restrictive or obstructive type, and pneumothorax
Cu IARC: group 3 (Gaetke et al., 2014; Barber et al., 2021; Royer and Sharman, 2023)	• ROS production/OxS damage, regulates lipid metabolism, antimicrobial defense, neuronal activity, resistance of tumor cells to chemotherapeutic drugs, kinase-mediated signal transduction, DNA damage, reduces cell proliferation, α-synuclein aggregation, activates acidic sphingomyelinase, and releases ceramide	• Induced increased lung weight, bronchoalveolar lavage fluid changes, alveolar histiocytosis, bronchioloalveolar hyperplasia, acute neutrophilic inflammation in the lungs, and nasal olfactory epithelial degeneration
Mn (O'Neal and Zheng, 2015; Evans and Masullo, 2023; Magro et al., 2025)	• ROS production/OxS damage, acts as a cofactor for many enzymes (e.g., MnSOD), involved in arginase activity in the urea cycle, regulates neurotransmitter synthesis (dopamine, glutamate, and GABA), and maintains neural signaling and survival	• Increased incidence of cough, bronchitis, and susceptibility to infectious lung disease, pneumonitis, and pneumonia
Ag (Ferdous and Nemmar, 2020; Steck and Murray, 2024)	• ROS production/OxS damage, interacts with respiratory chain proteins on the membrane, interrupts intracellular O2 reduction	• Pneumonitis, respiratory irritation, and tracheal epithelium structural damage
Sn (Toxicological Profile for NCBI, 2005; Granjeiro et al., 2020)	• Catalyzes the ROS production/OxS damage, modulates synaptic transmission, hyperglycemic toxicity, and produces degenerative lesions in the hippocampus and associated structures of the limbic system, stimulate the neuronal release of and/or decrease neuronal cell uptake of neurotransmitters (e.g., aspartate, GABA, glutamate, norepinephrine, and serotonin), decreases the expression of neural cell adhesion molecule (e.g., NCAM) and cell migration during the development of the nervous system, produces loss of hippocampal neurons, expression of several neuropeptides (e.g., dynorphin, enkephalin, neuropeptide Y, somatostatin), and neuropeptide receptors (e.g., neuropeptide Y), increases the expression of pro-inflammatory cytokines (e.g., tumor necrosis factor-, interleukin-1, and monocyte chemoattractant protein 1), induces a heat shock protein (HSP32) an indicator of OxS, increases apoptosis, suppresses the proliferation, cytotoxicity involves suppression of DNA and protein synthesis, suppresses the lymphoproliferative responses to T- and B-cell mitogens	• Benign pneumoconiosis (stannosis), discrete opaque shadows throughout the lungs, respiratory depression, producing irritation of the upper respiratory tract and chest irritation, tightness, and pain, sore throat, burning nose, and wheezing, cough, and difficulty in breathing, characterized by inspiratory discomfort, shortness of breath, and chest discomfort, nasal discharge, lung edema, and congestion
Mo IARC: group 2B (Jonmaire, 2015; NCBI, 2020)	• Critical components of enzymes (e.g., xanthine oxidase, sulfite oxidase, and aldehyde oxidase), high levels of Cu and S enhance the elimination of Mo by blocking a protein carrier and, therefore, inhibiting reuptake, protecting against poisoning by metals, such as Cu and Hg, and may have anticarcinogenic activity	• Reported dyspnea and cough, increased predicted forced expiratory volume in 1 s (FEV1), subclinical alveolitis, chronic exposure resulted in lesions in the nose, larynx, and lungs, in the nose, hyaline degeneration of the respiratory and olfactory epithelia, squamous metaplasia of the epiglottis, chronic inflammation, alveolar epithelial metaplasia, and histiocytic cellular infiltration
Zn (Plum et al., 2010; Mizuno and Kawahara, 2013; Chen et al., 2024; Vali et al., 2025)	• Catalyzes ROS production/OxS damage, endoplasmic reticulum (ER)-stress-related genes (e.g., GADD34, GADD45, and p8), and the Ca-related gene Arc (activity-related cytoskeleton protein), maintains homeostasis and neuronal function, and metallothioneins regulate Zn in relation to cell cycle regulation, cell proliferation, and apoptosis	• Metal fume fever is characterized by respiratory effects such as chest pain, cough, and dyspnea

## 5 Uptake and deposition airborne metals and metalloids in lungs

The principal mechanisms responsible for airborne particle deposition include inertial impaction, gravitational sedimentation, Brownian diffusion, turbulence, electrostatic precipitation, and interception (Darquenne, 2020). The nose filters particles of approximately 1 μm; particles with an aerodynamic diameter greater than 100 μm possess a low probability of entering the human respiratory tract; however, particles smaller than 50 μm frequently enter. The particles with an aerodynamic diameter greater than 10 μm are primarily deposited in the extrathoracic region and the particles less than 10 μm are deposited in the tracheobronchial airways or the alveolar region. Particles deposited in the larger bronchi are more likely to be transported to the pharynx by the mucociliary escalator and subsequently ingested into the gastrointestinal tract. The mechanisms of PM clearance are dependent upon the regions of the respiratory tract, and they include absorptive and non-absorptive processes. These mechanisms include sneezing, nose wiping or blowing, mucociliary transport, dissolution and absorption into the blood or lymph, and endocytosis by phagocytes or epithelial cells. All of these mechanisms remove PM deposited in the extrathoracic or tracheobronchial regions. The process of PM deposition in the alveolar region may result in three outcomes: dissolution and subsequent absorption into the blood or lymph, endocytosis by phagocytes or epithelial cells, or translocation into the systemic circulation (Yokel et al., 2006; Elder et al., 2015). According to Khan et al. (2024), the deposited mass of PM_2.5-10_ in different regions of the human respiratory tract was distributed as follows: 0.5% in the pulmonary region, 2% in the tracheobronchial region, and 97.5% in the extrathoracic region. In the case of the lobar region, a higher deposition rate was observed in the medial (53%) and inferior (8%) segments when compared to the superior (39%) segment. The distribution and accumulation of airborne metals and metalloids within the pleural space are governed by complex fluid dynamics, primarily regulated by lymphatic drainage and gravitational forces. The homeostasis of pleural fluid is contingent upon a delicate balance between the processes of capillary filtration and lymphatic removal. In this regard, diaphragmatic and parietal lymphatics function as the predominant clearance pathways (Solari et al., 2022). Furthermore, gravitational gradients and respiratory mechanics influence fluid distribution, thereby promoting preferential accumulation in dependent regions of the pleural cavity. Disruption of lymphatic drainage or an imbalance in the production-absorption dynamics can result in fluid and potentially airborne metals and metalloids accumulation (D'Agostino and Edens, 2023; Krishna et al., 2024). Metals accumulate in the human lung parenchyma primarily through the inhalation of PM, with differing distribution patterns for various elements. As Zhang et al. (2022) have demonstrated, research findings indicate that elements such as Al, Ba, Pb, Cr, Ni, Sn, and Sb have been observed to undergo both intrapulmonary diffusion and extrapulmonary transfer to the circulatory system and remote organs. It has been proposed that Ti and V are biochemically stable and highly retained in pulmonary particles. A comprehensive analysis of the distribution of metals and their structural characteristics within pulmonary particulate matter has the potential to provide invaluable insights into their toxicokinetics.

## 6 Identification of airborne metals and metalloids in PE

Initial pleural fluid analysis is a critical component for the evaluation of suspected MPE. The predominant manifestation of MPE is as exudates, frequently hemorrhagic, with mononuclear cell predominance. Basic biochemical parameters, including carcinoembryonic antigen (CEA), albumin gradient, total protein (TP), cholesterol levels, lactate dehydrogenase (LDH), glucose, adenosine deaminase, and pH, have been demonstrated to facilitate the differentiation of MPE from other potential etiologies and to provide prognostic information. Indeed, the combination of these biochemical parameters enhances the precision of PE analysis (Aleksiev et al., 2025). Recently, novel techniques have been explored for the early detection of LC using liquid biopsy. These techniques employ biomarkers, including circulating cell-free DNA, microRNA molecules, circulating tumor cells, tumor-derived exosomes, tumor-educated platelets, and volatile organic compounds (VOCs) (Habbab et al., 2025). Currently, the presence of HM in the PE of LC patients has not been considered a potential biomarker that could promote disease progression or cause, and that could alter normal lung conditions. However, it is imperative to enhance the sensitivity and specificity of biomarkers to ensure an accurate diagnosis of the disease.

Dines et al. (1972) conducted a study to evaluate the levels of metals in two groups of pleural fluid: 86 patients with benign disease and 116 patients with malignant disease. The findings indicated a correlation between Cu and pleural fluid protein in the benign group, while Zn and Fe each exhibited a weak relationship to protein. The positive correlation between Cu and Zn in the benign group is significant. However, the evidence for any such relationship in the malignant group is lacking. Similarly, Tekşen et al. (1996) found that the levels of Se, Cu, Zn, and Mg in the serum and pleural fluid of patients diagnosed with malignant diseases. The study included 19 patients with LC (squamous cell, small cell, or ADE) and 17 patients with non-malignant diseases, including tuberculosis, pneumonia, CHF, and chronic renal failure. Serum concentrations of Cu and Mg were found to be significantly elevated in both malignant and non-malignant patient groups when compared with control subjects. The serum/pleural fluid ratio of Zn was found to be significantly lower in patients with malignant effusions compared to those with benign conditions. Likewise, Domej et al. (1997) identified the concentrations of 14 trace elements (Ba, Ca, Cd, Co, Cs, Cu, Mg, Mn, Mo, Pb, Rb, Sn, Sr, Zn) in PE from 17 patients. The concentrations of the essential trace elements (Co, Cu, Mn, Mo, Sn, and Zn) in the effusions are, in general, lower (25%–55%) than in the corresponding serum. The concentrations of Cd in the effusions were found to be comparable to those observed in the serum of three patients, significantly lower than those detected in the serum of four patients, and notably higher in the serum of three patients. In general, the concentrations of Pb in the effusions were found to be considerably higher than those found in the serum.

Later, the same author, Domej et al. (2000a); Domej et al. (2000b) discovered that elevated concentrations of Zn were a distinguishing feature of thoracic empyema. A high degree of significance was observed for nine of the 28 pairs of clinical parameters, including TP, Cu, and Zn in the effusions and ceruloplasmin. Furthermore, the concentrations of elements ascertained in the empyema were often elevated in comparison to those observed in PE derived from other benign or malignant conditions, except for Cu. Additionally, Mg, Mn, and Rb concentrations in the empyema were found to be elevated in comparison to the corresponding serum concentrations. In addition, Demirpençe et al. (2016) observed that of 38 patients, no significant difference was found between MPE and BPE with respect to concentrations of Cr, Cu, Ni, Pb, Se, and Zn. In the same way, Bai et al. (2021), a study including 63 patients with LC and 31 patients with CHF, revealed a negative correlation between PM_10_, PM_2.5_, and NO_2_ with Al in the PE, as well as a positive correlation between PM_2.5_ and Zn in the PE. A rise in PM_2.5_ of 1 μg/m^3^ and an increase in Zn of 1 ng/mL were associated with an adjusted OR of 2.4 for PM_2.5_ and an adjusted OR of 1.0 for Zn, when considering LC. The findings indicate that increases in PM_2.5_ and Zn in the PE are associated with an elevated risk of MPE in LC patients, as evidenced by the adjusted ORs of 1.5 and 1.0, respectively. Furthermore, an increase of 1 μg/m^3^ in PM_2.5_ levels augmented the probability of developing ADE. The overall OR for patients diagnosed with LC was 1.7, indicating an approximate approach to treatment. Finally, Fitzgerald et al. (2021) identified MPE (58 patients), benign exudates (26 controls), and fluid overload (21 patients) in pleural fluid samples from 105 patients. Overall, the median concentrations of Cu and Mo were higher than the reference range for blood levels or within this range. The concentrations of Zn and Cu exhibited a marked increase in exudates compared to transudates, with respective elevations of 2.6- and 2.20-fold. Table 2 provides a comprehensive overview of the studies that have identified the presence of various metals in the PE of patients diagnosed with LC. The results indicated slight variations in the levels of metals or metalloids detected in MPE and BPE, with no significant differences observed across the various metals. Therefore, it is necessary to consider specific groups and exposure scenarios, along with lifestyle, work conditions, and geographical areas that may exhibit unique local circumstances. Furthermore, the collection of data from cohort studies and case-control research is essential to elucidate the correlation between the presence of metals or metalloids in PE and cancer. A comprehensive analysis should encompass a detailed assessment of the association of cancer risk factors and socioeconomic variables (Hamzah et al., 2016; Pinto et al., 2017; Bailey et al., 2025; Gonzalez-Villalva et al., 2026).

**TABLE 2:** Identification of airborne metals in MPE and BPE.

Author	Chemical compound	MPE	BPE	*P*
Dines et al. (1972) MPE (n = 116) BPE (n = 86) (USA)	Cu (µg/mL)	0.846 ± 0.046	0.693 ± 0.125	NR
	Zn (µg/mL)	0.452 ± 0.026	0.413 ± 0.155	NR
	Fe (µg/mL)	0.537 ± 0.030	0.555 ± 0.220	NR
Teksen et al. (1996) MPE (n = 19) BPE (n = 17) (Tukey)	Se (ng/mL)	43.08 ± 1.57	43.47 ± 16.15	>0.05
	Cu, µg/ml	0.75 ± 0.28	0.82 ± 0.38	>0.05
	Zn (µg/mL)	0.51 ± 0.1	0.44 ± 0.18	>0.05
	Mg (µg/mL)	23.99 ± 2.78	22.09 ± 2.95	>0.05
Domej et al. (2000a) MPE (n = 34) BPE (n = 27) (Austria)	Cu (µg/kg)	720 (220–1,520)	520 (58–1,390)	NR
	Zn (µg/kg)	410 (147–530)	300 (27–1,000)	NR
Demirpençe et al. (2016) MPE (n = 8) BPE (n = 30) (Turkey)	Cr (ng/mL)	43 ± 24.1	34.9 ± 14.7	0.28
	Cu (ng/mL)	0.99 ± 0.38	0.81 ± 0.32	0.20
	Ni (ng/mL)	58.1 ± 25.4	67 ± 21.7	0.32
	Pb (ng/mL)	107.3 ± 80.1	74.9 ± 34.2	0.28
	Se (ng/mL)	73.7 ± 38.4	68.2 ± 35	0.73
	Zn (ng/mL)	657.8 ± 205.3	615.5 ± 123.7	0.72
Bai et al. (2021) LC (n = 63) CHF (n = 31) (Taiwan)	PM_10_ (µg/m^3^)	40.0 ± 3.8	39.4 ± 4.3	NS
	PM_2.5_ (µg/m^3^)	25.7 ± 1.6	24.4 ± 1.3	<0.05
	NO_2_ (ppb)	20.3 ± 3.2	19.6 ± 3.4	NS
	SO_2_ (ppb)	3.2 ± 0.7	3.0 ± 0.7	NS
	Al (ng/mL)	272 ± 174	341 ± 214	NS
	Fe (ng/mL)	1799 ± 1758	1,356 ± 1,004	NS
	Cu (ng/mL)	786 ± 381	672 ± 282	NS
	Zn (ng/mL)	422 ± 223	314 ± 176	<0.05
	Pb (ng/mL)	3.1 ± 2.2	2.8 ± 1.6	NS
Fitzgerald et al. (2021) MPE (n = 58) BPE (n = 26) (Australia)	Zn (µmol/L)	6.4 (3.5–9.2)	7.55 (5.8–14.1)	<0.03
	Cu (µmol/L)	10.1 (6.0–16.4)	14.5 (9.3–18.1)	0.05
	Co (nmol/L)	1.6 (1.1–1.9)	2.6 (1.1–2.4)	<0.001
	Mn (nmol/L)	10.7 (5.5–17.6)	12.7 (9.5–21.51)	NR
	Fe (µmol/L)	11.1 (4.6–20.3)	5.8 (2.7–12.8)	0.07
	Mo (nmol/L)	7.0 (4.0–10.6)	11.9 (5.9–32.8)	0.01

The results indicate the mean ± standard deviation, the median value (minimum-maximum values), and a not-significant difference, *P* > 0.05 (NS), not reported (NR).

## 7 Association of OxS in PE induced by airborne metals and metalloids

The toxicity of HM is a significant threat to human health, with numerous associated health risks. This process can result in significant damage due to the generation of ROS that induce OxS in cells and tissues, as well as the capacity of a biological system to detoxify these reactive products. It has been demonstrated that ROS are capable of performing physiological functions, including cell signaling. Even if the generation of these by-products is a natural consequence of oxygen metabolism, an imbalance in ROS production has been observed to result from the interaction of environmental stressors (e.g., UV radiation, ionizing radiation, pollutants, and HM) and xenobiotics (e.g., antineoplastic drugs). This imbalance has the potential to induce damage to vital cellular components (Pizzino et al., 2017; Sies et al., 2017). As noted by Najeeb et al. (2012), a prospective study was conducted that involved 96 patients (48 cases of MPE, 48 cases of BPE). The pleural fluid levels of malondialdehyde (MDA), superoxide dismutase (SOD), LDH, and TP were elevated in MPE samples, exhibiting a mean level of 126.8 ± 12.3 nmol/mL, 38.6 ± 5.7 U/ml, 185.3 ± 21.5 IU/L, and 4.3 ± 0.8 gm%, respectively. In BPE, the mean levels were determined to be 53.0 ± 8.9 nmol/mL, 32.2 ± 3.3 U/ml, 120.3 ± 15.4 IU/L, and 4.1 ± 0.5 gm%, respectively. Furthermore, Yadav (2019) established that a total of 75 individuals were included, divided into two groups: transudate pleural effusion (21 patients) and exudative pleural effusion (54 patients). The uric acid, an indicator of OxS and a natural antioxidant, was lower in the exudative fluid compared to the transudate effusion. C-reactive protein (CRP) has been identified as a marker for infection and is categorized as an acute-phase protein. A substantial increase in the concentration of CRP was observed in exudative pleural fluid compared to transudate fluid. MDA has been identified as a marker for membrane lipid peroxidative damage resulting from local free radical generation in pleural fluid. The levels of MDA in exudative fluid were found to be significantly higher compared to transudate fluid. A positive correlation with CRP and a negative correlation with uric acid have been demonstrated, indicating that OxS is more prevalent in exudates than in transudates. A subsequent analysis by Papageorgiou et al. (2005) was conducted on a cohort of 90 patients with a final diagnosis of PE. The OxS were found to be elevated in exudates in comparison to transudates. The area under the ROC curve (AUC) was determined to be 0.9, and the method demonstrated high sensitivity (96.8%), high specificity (96.3%), and high accuracy (96.7%) for the diagnosis of exudates. Similarly, Cobanoglu et al. (2010) measured the reactive oxygen metabolites (ROM) in 60 subjects with exudative PE and 25 healthy individuals (control). The serum ROM values of patients with non-MPE were found to be significant when compared to the control group. Furthermore, a statistically significant discrepancy was identified between the ROM values of smokers and nonsmokers within each of the three groups.

Likewise, Reddy et al. (2009) conducted a study with 90 patients, who were divided into two groups. The initial cohort comprised 18 patients exhibiting fluid accumulation due to diverse underlying conditions, including heart failure, renal problems, and low protein levels. The second group comprised 72 patients with fluid buildup due to various underlying conditions, including tuberculosis, cancer, or lung infection. The study revealed that the level of a specific marker, as measured by MDA, was elevated in the second group compared to the first. Furthermore, the second group demonstrated an augmented level of another marker, designated as LDH, in comparison to the first group. In the same way, Ethemoğlu et al. (2025) found in 50 patients diagnosed with PE and 30 controls were examined total antioxidant capacity (TAC) measurements. The group exhibiting exudate and transudate demonstrated higher total oxidative status levels in comparison to the control group. The mean TAC level in the control group was found to be higher than in the exudate and transudate groups. The Reactive Oxygen Species Modulator 1 (ROMO1) is a nuclear-encoded inner mitochondrial protein that is recognized for its dual role as a modulator of ROS and a non-selective ion channel. The initial identification of ROMO1 was due to its role in ROS production. Subsequent studies have identified its role as a non-selective ion channel, responsible for maintaining ion homeostasis within the mitochondrial matrix (Yordanov and Tsoneva, 2025). As noted by Chen et al. (2017), a study was conducted on a cohort of 102 patients diagnosed with primary empyema; the concentrations of ROMO1 in pleural fluid and serum from patients diagnosed with MPE exhibited significantly higher levels in comparison to those observed in patients diagnosed with BPE. The diagnostic sensitivity and specificity of pleural fluid ROMO1 were 61.5% and 82.0%, respectively, but the diagnostic sensitivity and specificity of serum ROMO1 were only 41.4% and 86.2%, respectively. These findings suggest that serum ROMO1 may not be a reliable diagnostic tool for distinguishing MPE from BPE. The diagnosis of PE origin is complicated by the wide range of potential etiologies. Exudates are frequently observed in patients with LC, while transudates are characteristic of non-cancerous cases. Nevertheless, the risk of misclassification remains a leading concern. The utilization of OxS has the potential to serve as a distinct marker, facilitating the differentiation between exudate and transudate PE. Several studies have demonstrated that exudate effusions exhibit significantly elevated levels of OxS and diminished levels of the antioxidant system when compared to transudate effusions (Papageorgiou et al., 2005; Ethemoğlu et al., 2025). Consequently, it is required to first ascertain the origin of the PE, followed by the analysis of the concentrations of metals and metalloids. This facilitates the determination of the effect of metals on the generation of ROS/OxS and the antioxidant response.

## 8 Regulation of the response of the antioxidant system in PE

Antioxidant species have been demonstrated to facilitate the lungs in counteracting the detrimental effects of ROS/OxS. The normal human lung is characterized by its efficient protection and buffer function against exogenous free radicals. In addition to classical antioxidant enzymes (AOE), the epithelial lining fluid contains a variety of low-molecular-weight antioxidants and proteins, including a tripeptide (e.g., glutathione (GSH)), mucin glycoproteins, proteins capable of binding metals (e.g., transferrin, ferritin, ceruloplasmin, lactoferrin), lipids (e.g., vitamin E), and water-soluble vitamins (e.g., vitamin C). The major human AOE include SODs, catalase (CAT), and enzymes associated with GSH metabolism and its synthesis, such as glutathione peroxidases (GPx) and γ-glutamyl cysteinyl synthetase (γGCS) (Rahman et al., 2006). According to Durak et al. (1996), the serum of 25 control subjects, as well as the activity of the SODs in the serum and pleural fluid samples from patients suffering from LC, tuberculosis, CHF, and from control subjects, was examined. Serum activities of SOD enzymes were found to be elevated in patient groups in comparison to control groups. The highest enzyme activities were measured in the serum and pleural fluids of the tuberculosis group. A weak correlation was observed between cytoplasmic and mitochondrial SOD activities in serum from the control group. Similarly, Wang et al. (2014) reported that the concentrations of MnSOD were determined in PE from 54 patients with tuberculous pleural effusion and 33 with MPE. Levels of the MnSOD enzyme were found to be significantly elevated in patients diagnosed with tuberculous pleural effusion, in contrast with those diagnosed with MPE. Likewise, Zalewska-Ziob et al. (2019) examined a sample of 53 individuals and found that the activity of antioxidant enzymes, including SOD with different isoforms (e.g., Cu/ZnSOD and MnSOD), CAT, glutathione reductase (GR), glutathione S-transferase (GST), and MDA, differed between tumor and adjacent non-cancerous tissues of two histological types of non-small cell lung cancer (ADE and squamous cell carcinoma). The concentration of MDA was found to be comparable in tumors and adjacent non-cancerous tissues. The antioxidant response of the lungs to the chronic release of ROS, as occurs in the immune-specific granulomatous inflammation of chronic beryllium disease (CBD). The AOE (SOD, CAT, GPx, and GSH) were quantitatively analyzed in the lung epithelial lining fluid (ELF) and serum of control subjects, cigarette smokers, and individuals with CBD. An increase in GPx activity and extracellular GPx (eGPx) protein in the ELF of subjects with CBD was observed in comparison with that of control subjects and smokers. A positive correlation was identified between increased GSH and eGPx levels. This finding suggests that the induction of these antioxidants is attributable to analogous mechanisms (Comhair et al., 1999). Thiol, an organic compound, contains a sulfhydryl group, which is critical for cells to manage OxS. The sulfhydryl groups found in sulfur-containing amino acids, such as cysteine and methionine, in proteins, are the primary targets of ROS. In the presence of ROS, in conjunction with those found in small molecules, cysteine residues, and other thiol-containing structures, undergo oxidation within the cell. This oxidation process leads to the formation of reversible disulfide bonds. Subsequent to this, these bonds can be broken down back into thiol groups, thereby illustrating the mechanism underlying thiol/disulfide balance. Recent studies have demonstrated that OxS in cancer cells results in a greater degree of redox imbalance compared to healthy cells. The significance of the dynamic thiol/disulfide balance in numerous biological processes, including antioxidant protection, detoxification, cell death, enzyme activity regulation, gene expression, and cell signaling (Poole, 2015).

As explained by Şener et al., 2020, found in a prospective, controlled, non-blinded study, participants comprised healthy volunteers and patients diagnosed with LC who had not yet initiated any treatment. The patient cohort included 45 male patients (90%) and five female patients (5%), while the healthy volunteer cohort included 41 male volunteers (82%) and nine female volunteers (18%). A general finding of the study was that the thiol levels in patients were lower than in the control group. A substantial relationship was identified between LC and thiol/disulfide homeostasis (TDH); however, no correlations were observed between disease stage and clinical performance status. In addition, Gormeli-Kurt et al. (2021) developed a prospective study including 50 patients with PE, who decompensated heart failure (56%), hepatic cirrhosis (36%), and hypoalbuminemia (8%). In the cohort of patients diagnosed with exudative PE, 17 patients (68%) were found to have malignancy, 7 patients (28%) had parapneumonic effusion, and 1 patient (4%) had a pulmonary embolism. Moreover, the study was stratified into two groups: transudate PE and exudate PE. A substantial decrease in total thiol (TT) and native thiol (NT) values was observed in the transudative PE group in comparison with the exudative PE group. However, no significant discrepancy was observed between the two groups with respect to the disulfide levels. In the same way, Abuzaina et al. (2022) found that the dynamic equilibrium between thiol and disulfide groups in100 patients diagnosed with PE. Levels of disulfide (D), TT, and NT were found to be significantly higher in the exudative group than in the transudative group. The ratio of D/NT and D/TT was found to be higher in the transudative group. The findings indicated the presence of elevated levels of NT and TT in the exudates, suggesting a heightened state of OxS in exudative PE.

The expression of MnSOD in normal lung tissue and in bronchial, alveolar, and alveolar macrophages is low. The elevated levels of the enzyme MnSOD in malignant pleural mesothelioma are a reliable indicator of a poor prognosis and a high resistance to chemotherapies and radiation. Cu/ZnSOD, a cytosolic enzyme, is expressed in bronchial epithelium and is relatively constitutive. In the lung, the presence of CAT is predominantly localized in alveolar macrophages and alveolar epithelium. It can be hypothesized that thiol proteins play a fundamental role in carcinogenesis and tumor progression (Kinnula and Crapo, 2003). As illustrated in Table 3, the studies encompassing the levels of OxS biomarkers and the enzymes associated with the antioxidant response in MPE in patients diagnosed with LC.

**TABLE 3:** Biomarkers of OxS and antioxidant enzymes response in MPE and BPE.

Author	Antioxidant unzymes	MPE	BPE	*P*
Durak et al. (1996) MPE (n = 17), BPE (n = 25), (Turkey)	Cu/Zn SOD (U/ml)	4.42 ± 2.69	NS (No sample)	<0.0005
	Mn SOD (U/ml)	3.43 ± 1.32	NS (No sample)	<0.0005
	Total SOD (U/ml)	7.8 ± 2.4	NS (No sample)	<0.0005
Domej et al. (2000a) MPE (n = 34), BPE (n = 27), (Austria)	LDH (U/L)	235 (48–1,595)	160 (49–642)	NR
	CRP (mg/L)	15 (0–160)	24.0 (0.178)	NR
Najeeb et al. (2012) MPE (n = 48), BPE (n = 48), (India)	MDA (nmol/mL)	126.8 ± 12.3	53.0 ± 8.9	<0.001
	SOD (U/ml)	38.6 ± 5.7	32.2 ± 3.3	<0.001
	LDH (IU/L)	185.28 ± 21.48	120.3 ± 15.4	<0.001
	TPR (gm %)	4.34 ± 0.84	4.12 ± 0.47	0.122
Wang et al. (2021) MPE (n = 54), MPE (n = 33), (China)	Mn SOD (ng/mL)	42.1 (34.4–56.9)	73.5 (64.7–82.9)	<0.05
Lee et al. (2017) MPE (n = 53), BPE (n = 91), (Republic of Korea)	ROMO1 (ng/mL)	99.3 (50.9–169.6)	34.1 (22.8–58.1)	<0.001)
Zhang Z. et al. (2020) MPE (n = 75), BPE (n = 41), (China)	ROMO1 (ng/mL)	41.3 (5.2–334.2)	16.6 (1.4–67.2)	<0.0001
	Ferritin (μg/L)	1,328.6 (51.4–12527.0)	897.4 (367.6–6899.0)	<0.0001
	LDH (U/ml)	382.0 (34.2–5663.0)	404.0 (127.0–1819.0)	<0.0001
Bai et al. (2021) LC (n = 63), CHF (n = 31), (Taiwan)	LDH (U/L)	403 ± 573	127 ± 100	<0.05
Gormeli-Kurt et al. (2021) (Exudate = 25), (Transudate = 25), (Turkey)	NT (μmol/L)	Exudate 369.4 ± 51.0	Transudate 422.2 ± 40.4	<0.0001
	TT (μmol/L)	Exudate 411.9 ± 56.1	Transudate 462.0 ± 41.1	<0.001
	D (μmol/L)	Exudate 21.21 ± 6.67	Transudate 19.94 ± 6.25	0.489
Abuzaina et al. (2022) (Exudate = 50), (Transudate = 50), (Turkey)	NT (μmol/L)	Exudate 119.1 ± 72.1	Transudate 38.85 ± 36.8	<0.0001
	TT (µmol/L)	Exudate 14,605 ± 76.5	Transudate 50.0 ± 44.9	<0.0001
	D (µmol/L)	Exudate 13.0 ± 6.1	Transudate 6.12 ± 5.3	<0.0001

The results indicate the mean ± standard deviation, the median value (minimum-maximum values), and a not-significant difference, P > 0.05 (NS), not reported (NR).

## 9 Airborne metals and metalloids induces ROS/OxS in the pleural space

Normal cells possess complex biochemical and genetic mechanisms to maintain ROS balance, and their perturbation can have pathophysiological consequences. Cancer cells exhibit aberrant redox homeostasis. While ROS are pro-tumorigenic, in normal cells, high ROS levels are cytotoxic (Hayes et al., 2020). A limitation of this study is the inherent difficulty of distinguishing endogenous from exogenous sources of ROS in the pleural fluid microenvironment. Tumor cells are characterized by elevated basal ROS levels, resulting from their metabolic activity, mitochondrial dysfunction, and oncogene-driven signaling. These factors, in combination, amplify ROS production via two primary mechanisms: the mitochondrial electron transport chain and the NADPH oxidase system (Ghoneum et al., 2020). The process of metal carcinogenesis occurs through the involvement of two distinct stages. In the initial stage of metal-induced carcinogenesis or cell transformation, elevated levels of ROS act as oncogenes, inducing DNA damage, impeding DNA repair, and perturbing normal signal transduction pathways. The culmination of these events invariably gives rise to malignant transformation. In the second stage of metal carcinogenesis, metal-transformed cells with low levels of ROS exhibit carcinogenic properties by promoting apoptosis resistance. Concurrently, exogenous factors such as PM_2.5_-associated metals present in pleural fluid can independently catalyze ROS generation (Xu et al., 2017). A finding is the convergence of both sources on overlapping molecular pathways, resulting in the production of indistinguishable ROS species. This phenomenon is further compounded by the fact that cancer cells already function in a state of elevated OxS, which can be exacerbated by additional exogenous insults (Jomova et al., 2025). Therefore, the inability to definitively distinguish whether ROS arise predominantly from tumor-intrinsic metabolism or from PM_2.5_-metal exposure represents a confounding factor. This limitation significantly constrains causal inference, as OxS in the pleural space likely reflects a composite signal arising from both intrinsic tumor biology and environmental exposures. Consequently, any interpretation associating PM_2.5_-metals with oxidative damage should be situated within a theoretical framework that incorporates additive or synergistic redox interactions, as opposed to asserting direct causality. This conceptual limitation highlights the necessity for subsequent studies to incorporate source-specific probes, metal chelation strategies, or isotopic tracing approaches. These methods are necessary for resolving the relative contributions of endogenous and exogenous ROS in the tumor-associated pleural environment.

**FIGURE 2:**
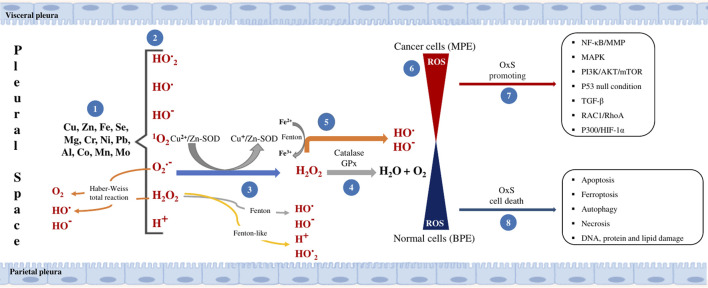
Mechanism of ROS generation and OxS by PM_2.5_. The presence of airborne metals and metalloids in the pleural space has been observed to be associated with cancer progression and normal cell response. Adapted from Wang et al. (2021), Ma et al. (2025); Olazábal-Morán et al. (2025).

The presence of these metals and metalloids in PE suggests a potential role in the progression of LC, due to their capacity to influence the balance of the antioxidant system and, consequently, regulate diverse signaling pathways (Figure 2). As indicated (1), the presence of some metals or metalloids in PE associated with anthropogenic emissions has been reported. Despite the scarcity of reports on the identification of some of these substances, it is essential to expand the characterization of a greater number to determine their potential health impact. It has been demonstrated that the presence of certain metals can elicit the generation of ROS. (2) However, it is imperative to consider the potential interaction between other contaminants and reactive species, such as nitrogen, non-radical products like H_2_O_2_ or hypochlorous acid, potent oxidizing agents. (3) The antioxidant system response must maintain the balance between ROS generation and elimination; however, there is a paucity of reports focusing on identifying the activity of antioxidant enzymes in PE. (4) A decrease in Cu/Zn-SOD activity has been identified in MPE compared to BPE samples. It is necessary to identify the presence and role of other enzymes, such as CAT or GPx. (5) The oxidation reactions, including the Fenton reaction, the Fenton-like reaction, and the Haber-Weiss reaction. These reactions have been demonstrated to sustain a steady rate of ROS generation in the presence of metals. (6) It has been demonstrated that ROS plays a pivotal role as a mediator in cancer development. This molecule exhibits the capacity to both promote and protect against tumorigenesis. In the context of cancer cells, the presence of ROS participates in the initiation, promotion, and progression of the disease, inducing the activation of oncogenes, the inactivation of tumor suppressor genes, or genomic instability. Conversely, a substantial accumulation of ROS in normal cells induces cell death pathways. (7) In the context of cancer, the ROS have been observed to regulate various signaling pathways, including NF-κB/MMP, MAPKPI3K/AKT/mTOR, P53 null, TGF-β, RAC1/RhoA, and P300/HIF-1α. These pathways have been implicated in a number of processes, including cell proliferation, survival, and metastasis. (8) In typical circumstances, elevated levels of ROS have been demonstrated to induce OxS, resulting in a series of cellular responses, including apoptosis, ferroptosis, autophagy, necrosis, and damage to DNA, proteins, and lipids. This sequence of events has the capacity to inhibit tumorigenesis (Wang et al., 2021; Ma et al., 2025; Olazábal-Morán et al., 2025).

## 10 Implications airborne metals and metalloids toxicokinetic and toxicodynamic

The structural and functional similarities to metals and metalloids are transported and eliminated by cellular mechanisms. The molecular mimicry mechanism, in which metals bind to nucleophilic sites on certain biological molecules. The complexes formed can “mimic” the structural and functional endogenous substrates that normally bind to, or occupy, the active sites of carrier proteins, channels, structural proteins, enzymes, and/or transcription factors. The ionic mimicry mechanism describes the ability of an unbound, cationic species of a metal to behave or serve as a structural and functional homolog or mimic of another element at the site of a carrier protein, ion channel, enzyme, structural protein, transcription factor, and metal-binding protein (Bridges and Zalups, 2005). Consequently, the absorption, elimination, and toxicokinetics of these metals should be considered to be highly correlated for exposed individuals. This phenomenon can be attributed to the susceptibility of individuals to lung diseases, which results in differential effects of multiple metals. In instances where susceptible individuals are exposed to combinations of metals while demonstrating high absorption, there is an increased probability of toxic effects, either due to additive or synergistic interactions. Indeed, these metals have the potential to accumulate in the liver and kidneys. Due to their extended half-lives, these substances have the capacity to affect the hepatic and renal levels of Cytochrome P450 (CYP450) enzymes. As a result, it is essential to establish a framework that connects the toxicokinetics of metals, the dose-response relationships of CYP450, and the resulting effects of metals on the toxicokinetics of other elements (Sasso et al., 2010). The metal species (isotopic composition, electronic configuration, oxidation state, or molecular arrangement) can significantly affect their toxicokinetics and toxicodynamics, which in turn affect human health. This impact occurs through various mechanisms, including the transport of specific metal types via carrier membranes, the oxidation state, size of particles, the types of ligands that bind to metals, the differentiation between organic and inorganic metal forms, and biotransformation processes of metal species (Yokel et al., 2006).

## 11 Conclusions and perspectives

PM_2.5_ is a heterogeneous mixture of complex components from multiple sources, producing chemical and biological toxicities. The surface of PM_2.5_ demonstrates a tendency to adsorb metals and metalloids, a phenomenon attributable to the intrinsic properties. The result of this process is the potential for the development of pulmonary diseases, including LC. The presence of air pollution metals gives rise to distinctive physicochemical characteristics and properties that contribute to their toxicity. An increase in ROS/OxS is a fundamental aspect of the toxicity and carcinogenicity of these metals. The findings revealed a higher concentration of Zn in MPE compared to BPE, a statistically significant difference. Furthermore, an elevated concentration of trace elements, including Cu, Cr, Pb, Se, and Fe, has been documented in MPE. In the context of the antioxidant response, an increase in SOD was observed in MPE, as well as in the oxidative stress markers LDH, MDA, and ROMO1. In this review, several limitations are identified, including the limited number of studies that have examined the presence of metals and metalloids from air pollution identified in MPE, particularly those directly associated with ROS/OxS generation. Moreover, the studies have included a modest number of patients, making it recommended that subsequent studies consider the number of participants in their design. Consequently, it is imperative to identify a greater number of metals, particularly those that predominantly contain PM_2.5_. These findings indicate that the identification of antioxidants and OxS biomarker is constrained. It is imperative to ascertain the contributions of additional components, including carbon components, PAHs, VOCs, and toxins, in the generation of ROS/OxS.

## References

[B1] Abuzaina O. Hezer H. Karalezli A. Abuzaina S. Neşelioğlu S. Erel O. (2022). The role of Thiol/Disulfide homeostasis in the differentiation of transudative and exudative pleural effusion, running title: pleural fluid of Thiol/Disulfide homeostasis. Ann. Med. Res. 29 (2), 118–123. 10.5455/annalsmedres.2021.02.162

[B2] Aleksiev V. Markov D. Bechev K. (2025). Tumor markers in pleural fluid: a comprehensive Study on diagnostic accuracy. Diagnostics 15 (2), 204. 10.3390/diagnostics15020204 39857088 PMC11765104

[B3] Alves C. Evtyugina M. Vicente E. Vicente A. Rienda I. C. de la Campa A. S. (2023). PM2.5 chemical composition and health risks by inhalation near a chemical complex. J. Environ. Sci. (China). 124, 860–874. 10.1016/j.jes.2022.02.013 36182189

[B4] Bai K. J. Ho S. C. Tsai C. Y. Chen J. K. Lee C. N. Lee K. Y. (2021). Exposure to PM2.5 is associated with malignant pleural effusion in lung cancer patients. Ecotoxicol. Environ. Saf. 208, 111618. 10.1016/j.ecoenv.2020.111618 33396138

[B5] Bailey J. McFarlane S. Amarakoon I. (2025). Heavy metal carcinogenicity: a scoping review of observational & experimental evidence. Front. Oncol. 15, 1569816. 10.3389/fonc.2025.1569816 40860840 PMC12375472

[B6] Barber R. G. Grenier Z. A. Burkhead J. L. (2021). Copper toxicity is not just oxidative damage: zinc systems and insight from Wilson disease. Biomedicines 9 (3), 316. 10.3390/biomedicines9030316 33804693 PMC8003939

[B7] Bashour S. I. Mankidy B. J. Lazarus D. R. (2022). Update on the diagnosis and management of malignant pleural effusions. Respir. Med. 196, 106802. 10.1016/j.rmed.2022.106802 35287006

[B8] Bolan N. Kumar M. Singh E. Kumar A. Singh L. Kumar S. (2022). Antimony contamination and its risk management in complex environmental settings: a review. Environ. Int. 158, 106908. 10.1016/j.envint.2021.106908 34619530

[B9] Bonfiglio R. Scimeca M. Mauriello A. (2023). The impact of aluminum exposure on human health. Arch. Toxicol. 97 (11), 2997–2998. 10.1007/s00204-023-03581-6 37597077

[B10] Boreiko C. J. Rossman T. G. (2020). Antimony and its compounds: health impacts related to pulmonary toxicity, cancer, and genotoxicity. Toxicol. Appl. Pharmacol. 403, 115156. 10.1016/j.taap.2020.115156 32710957

[B123] Bray F. Ferlay J. Soerjomataram I. Siegel R. L. Torre L. A. Jemal A. (2018). Global cancer statistics 2018: GLOBOCAN estimates of incidence and mortality worldwide for 36 cancers in 185 countries. CA Cancer J. Clin. 68 (6), 394–424. 10.3322/caac.21492 30207593

[B11] Bridges C. C. Zalups R. K. (2005). Molecular and ionic mimicry and the transport of toxic metals. Toxicol. Appl. Pharmacol. 204 (3), 274–308. 10.1016/j.taap.2004.09.007 15845419 PMC2409291

[B12] Catalani S. Rizzetti M. C. Padovani A. Apostoli P. (2012). Neurotoxicity of cobalt. Hum. Exp. Toxicol. 31 (5), 421–437. 10.1177/0960327111414280 21729976

[B13] Cha Z. Zhang X. Zhang K. Zhou G. Gao J. Sun S. (2025). Atmospheric heavy metal pollution characteristics and health risk assessment across various type of cities in China. Toxics 13 (3), 220. 10.3390/toxics13030220 40137547 PMC11945492

[B14] Charkiewicz A. E. Omeljaniuk W. J. Nowak K. Garley M. Nikliński J. (2023). Cadmium toxicity and health Effects-A brief summary. Molecules 28 (18), 6620. 10.3390/molecules28186620 37764397 PMC10537762

[B15] Charkiewicz A. E. Omeljaniuk W. J. Garley M. Nikliński J. (2025). Mercury exposure and health effects: what do we really know? Int. J. Mol. Sci. 26 (5), 2326. 10.3390/ijms26052326 40076945 PMC11899758

[B16] Chen R. J. Lee V. R. (2026). Cobalt toxicity. Treasure Island (FL): StatPearls Publishing. Available online at: https://www.ncbi.nlm.nih.gov/books/NBK587403/ (Accessed January 10, 2026). 36508548

[B17] Chen X. Zhang N. Dong J. Sun G. (2017). Reactive oxygen species modulator 1, a novel protein, combined with carcinoembryonic antigen in differentiating malignant from benign pleural effusion. Tumour Biol. 39 (5), 1010428317698378. 10.1177/1010428317698378 28459208

[B18] Chen P. Bornhorst J. Diana-Neely M. Avila D. S. (2018). Mechanisms and disease pathogenesis underlying metal-induced oxidative stress. Oxid. Med. Cell. Longev. 2018, 7612172. 10.1155/2018/7612172 30319733 PMC6167561

[B19] Chen B. Yu P. Chan W. N. Xie F. Zhang Y. Liang L. (2024). Cellular zinc metabolism and zinc signaling: from biological functions to diseases and therapeutic targets. Signal Transduct. Target Ther. 9 (1), 6. 10.1038/s41392-023-01679-y 38169461 PMC10761908

[B20] Cobanoglu U. Sayir F. Mergan D. (2010). Reactive oxygen metabolites can be used to differentiate malignant and non-malignant pleural effusions. Ann. Thorac. Med. 5 (3), 140–144. 10.4103/1817-1737.65042 20835307 PMC2930651

[B21] Comhair S. A. Lewis M. J. Bhathena P. R. Hammel J. P. Erzurum S. C. (1999). Increased glutathione and glutathione peroxidase in lungs of individuals with chronic beryllium disease. Am. J. Respir. Crit. Care Med. 159 (6), 1824–1829. 10.1164/ajrccm.159.6.9810044 10351926

[B22] D'Agostino H. P. Edens M. A. (2023). Physiology, pleural fluid. Treasure Island (FL): StatPearls Publishing. Available online at: https://www.ncbi.nlm.nih.gov/books/NBK513353/ (Accessed January 12, 2026). 30020725

[B23] Darquenne C. (2020). Deposition mechanisms. J. Aerosol. Med. Pulm. Drug Deliv. 33 (4), 181–185. 10.1089/jamp.2020.29029.cd 32598200

[B24] Demirpençe Ö. Mustafa Y. Alper A. Ersin K. Havin B. Hülya G. D. (2016). The role of trace elements in the malignant-benign differentation of pleural effusions. J. Clin. Anal. Med. 7, 364–367. 10.4328/JCAM.3366

[B25] Dines D. E. Elveback L. R. McCall J. T. (1972). Zinc, copper, and iron content of pleural fluid in benign and neoplastic disease. Thorax 27 (3), 368–370. 10.1136/thx.27.3.368 5039453 PMC472601

[B26] Domej W. Krachler M. Schlagenhaufen C. Trinker M. Krejs G. J. Irogolic K. J. (1997). Trace elements in pleural effusions. J. Trace. Elem. Med. Biol. 11 (4), 232–238. 10.1016/s0946-672x(97)80018-1 9575474

[B27] Domej W. Krachler M. Goessler W. Maier A. Irgolic K. J. (2000a). Pleural effusions and sera from patients with benign or malignant diseases. Biol. Trace Elem. Res. 78 (1-3), 13–33. 10.1385/bter:78:1-3:13 11314972

[B28] Domej W. Krachler M. Goessler W. Maier A. Irgolic K. J. Lang J. K. (2000b). Concentrations of copper, zinc, manganese, rubidium, and magnesium in thoracic empyemata and corresponding sera. Biol. Trace Elem. Res. 78 (1-3), 53–66. 10.1385/BTER:78:1-3:53 11314988

[B29] Durak I. Canbolat O. Kavutçu M. Oztürk H. S. Yurtarslani Z. (1996). Activities of total, cytoplasmic, and mitochondrial superoxide dismutase enzymes in sera and pleural fluids from patients with lung cancer. J. Clin. Lab. Anal. 10 (1), 17–20. 10.1002/(SICI)1098-2825(1996)10:1<17::AID-JCLA4>3.0.CO;2-I 8926562

[B30] Elder A. Nordberg G. F. Kleinman M. (2015). “Routes of exposure, dose, and toxicokinetics of Metals This chapter is based on the chapter routes of exposure, dose, and metabolism of metals,” in Handbook on the toxicology of metals Editors Beckett W. S. Nordberg G. F. Clarkson T. W. (Elsevier). 10.1016/B978-0-444-59453-2.00003-2

[B31] Ethemoğlu G. Gencer M. Aksoy N. (2025). Comparison of oxıdative and antioxidative parameters in the exudative and transudative pleural effusion. İJCMBS 5 (2), 52–60. 10.5281/zenodo.15618890

[B32] Evans G. R. Masullo L. N. (2023). “Manganese toxicity,” in StatPearls (Treasure Island (FL): StatPearls Publishing). Available online at: https://www.ncbi.nlm.nih.gov/books/NBK560903/ (Accessed January 20,2026). 32809738

[B33] Ferdous Z. Nemmar A. (2020). Health impact of silver nanoparticles: a review of the biodistribution and toxicity following various routes of exposure. Int. J. Mol. Sci. 21 (7), 2375. 10.3390/ijms21072375 32235542 PMC7177798

[B34] Fitzgerald D. B. Popowicz N. D. Joseph J. Butcher S. C. Westcott M. Lim E. M. (2021). Trace element levels in pleural effusions. Health Sci. Rep. 4 (2), e262. 10.1002/hsr2.262 33977154 PMC8093853

[B35] Gaetke L. M. Chow-Johnson H. S. Chow C. K. (2014). Copper: toxicological relevance and mechanisms. Arch. Toxicol. 88 (11), 1929–1938. 10.1007/s00204-014-1355-y 25199685 PMC4339675

[B36] Gates A. Jakubowski J. A. Regina A. C. (2023). “Nickel toxicology,” in StatPearls (Treasure Island (FL): StatPearls Publishing). Available online at: https://www.ncbi.nlm.nih.gov/books/NBK592400/ (Accessed January 21, 2026). 37276284

[B37] Gbondo D. Cerpa-Perez V. Pham N. M. Zhao Y. Rumchev K. (2025). Exposure to airborne contaminants and respiratory health among lithium mine workers in Western Australia. Environments 12 (6), 206. 10.3390/environments12060206

[B38] Genchi G. Carocci A. Lauria G. Sinicropi M. S. Catalano A. (2020). Nickel: human health and environmental toxicology. Int. J. Environ. Res. Public Health. 17 (3), 679. 10.3390/ijerph17030679 31973020 PMC7037090

[B39] Ghoneum A. Abdulfattah A. Y. Warren B. O. Shu J. Said N. (2020). Redox homeostasis and metabolism in cancer: a complex mechanism and potential targeted therapeutics. Int. J. Mol. Sci. 21 (9), 3100. 10.3390/ijms21093100 32354000 PMC7247161

[B40] Gonzalez-Villalva A. Marcela R. L. Nelly L. V. Patricia B. N. Guadalupe M. R. Brenda C. T. (2025). Lead systemic toxicity: a persistent problem for health. Toxicology 515, 154163. 10.1016/j.tox.2025.154163 40286900

[B41] Gonzalez-Villalva A. Rojas-Lemus M. López-Valdez N. Cervantes-Valencia M. E. Guerrero-Palomo G. Casarrubias-Tabarez B. (2026). Metal pollution in the air and its effects on vulnerable populations: a narrative review. Int. J. Mol. Sci. 27 (2), 720. 10.3390/ijms27020720 41596374 PMC12840800

[B42] Gormeli-Kurt N. Gokhan S. Erel O. Gunes C. Kahraman A. F. Ozhasenekler A. (2021). The role of pleural fluid thiol/disulphide homoeostasis in the differentiation between transudative and exudative pleural effusions. Int. J. Clin. Pract. 75 (4), e14051. 10.1111/ijcp.14051 33492739

[B43] Gouveia N. Rodriguez-Hernandez J. L. Kephart J. L. Ortigoza A. Betancourt R. M. Sangrador J. L. (2024). Short-term associations between fine particulate air pollution and cardiovascular and respiratory mortality in 337 cities in Latin America. Sci. Total Environ. 920, 171073. 10.1016/j.scitotenv.2024.171073 38382618 PMC10918459

[B44] Granjeiro J. M. Cruz R. Leite P. E. Gemini-Piperni S. Boldrini L. C. Ribeiro A. R. (2020). Health and environment perspective of tin nanocompounds: a safety approach. Tin Oxide Materials, 133–162. 10.1016/b978-0-12-815924-8.00006-2

[B45] Habbab F. M. Bédard E. L. R. Joy A. A. Alam Z. Abraham A. G. Roa W. H. Y. (2025). Early detection of lung cancer: a review of innovative milestones and techniques. J. Clin. Med. 14 (21), 7812. 10.3390/jcm14217812 41227214 PMC12609116

[B46] Haidar Z. Fatema K. Shoily S. S. Sajib A. A. (2023). Disease-associated metabolic pathways affected by heavy metals and metalloid. Toxicol. Rep. 10, 554–570. 10.1016/j.toxrep.2023.04.010 37396849 PMC10313886

[B47] Hamzah N. A. Mohd-Tamrin S. B. Ismail N. H. (2016). Metal dust exposure and lung function deterioration among steel workers: an exposure-response relationship. Int. J. Occup. Environ. Health. 22 (3), 224–232. 10.1080/10773525.2016.1207040 27392157 PMC5102237

[B48] Hasegawa S. (2022). Experimental characterization of PM2.5 organic carbon by using carbon-fraction profiles of organic materials. Asian J. Atmos. Environ. 16, 2021128. 10.5572/ajae.2021.128

[B49] Hayes J. D. Dinkova-Kostova A. T. Tew K. D. (2020). Oxidative stress in cancer. Cancer Cell 38 (2), 167–197. 10.1016/j.ccell.2020.06.001 32649885 PMC7439808

[B50] Hedya S. A. Avula A. Swoboda H. D. (2023). Lithium toxicity. Treasure Island (FL): StatPearls Publishing; Available online at: https://www.ncbi.nlm.nih.gov/books/NBK499992/ (Accessed January 21, 2026). 29763168

[B51] Ho S. C. Chuang K. J. Lee K. Y. Chen J. K. Wu S. M. Chen T. T. (2019). Chronic obstructive pulmonary disease patients have a higher risk of occurrence of pneumonia by air pollution. Sci. Total Environ. 677, 524–529. 10.1016/j.scitotenv.2019.04.358 31063895

[B52] Hong Y. S. Song K. H. Chung J. Y. (2014). Health effects of chronic arsenic exposure. J. Prev. Med. Public Health 47 (5), 245–252. 10.3961/jpmph.14.035 25284195 PMC4186552

[B53] IARC, Working Group on the Evaluation of Carcinogenic Risks to Humans (2016). “Outdoor air pollution,” in International agency for research on cancer (IARC monographs on the evaluation of carcinogenic risks to humans, no. 109.) 1.2, sources of air pollutants (Lyon (FR)). Available online at: https://www.ncbi.nlm.nih.gov/books/NBK368029/ (Accessed January 22, 2026).

[B54] Jaishankar M. Tseten T. Anbalagan N. Mathew B. B. Beeregowda K. N. (2014). Toxicity, mechanism and health effects of some heavy metals. Interdiscip. Toxicol. 7 (2), 60–72. 10.2478/intox-2014-0009 26109881 PMC4427717

[B55] Jany B. Welte T. (2019). Pleural effusion in adults-etiology, diagnosis, and treatment. Dtsch. Arztebl. Int. 116 (21), 377–386. 10.3238/arztebl.2019.0377 31315808 PMC6647819

[B56] Jomova K. Alomar S. Y. Nepovimova E. Kuca K. Valko M. (2025). Heavy metals: toxicity and human health effects. Arch. Toxicol. 99 (1), 153–209. 10.1007/s00204-024-03903-2 39567405 PMC11742009

[B57] Jonmaire P. (2015). Molybdenum. Hamilton and Hardy’s industrial toxicology. John Wiley & Sons, Inc., 167–172. 10.1002/9781118834015.ch24

[B58] Kebe M. Traore A. Sow M. Fall S. Tahri M. (2025). Human health risk evaluation of particle air pollution (PM10 and PM2.5) and heavy metals in Dakar's two urban areas. Asian J. Atmos. Environ. 19, 7. 10.1007/s44273-025-00056-1

[B59] Khan S. Veerendra S. Nachimuthu-Manoj K. Bhola-Ram G. (2024). Particulate matters deposition in the human respiratory system: a health risk assessment at a technical university. J. Air pollut. Health 9 (1), 1–14. 10.18502/japh.v9i1.15075

[B60] Kinnula V. L. Crapo J. D. (2003). Superoxide dismutases in the lung and human lung diseases. Am. J. Respir. Crit. Care Med. 167 (12), 1600–1619. 10.1164/rccm.200212-1479SO 12796054

[B61] Kopcinovic L. M. Culej J. (2014). Pleural, peritoneal and pericardial effusions - a biochemical approach. Biochem. Med. Zagreb. 24 (1), 123–137. 10.11613/BM.2014.014 24627721 PMC3936968

[B62] Krishna R. Antoine M. H. Alahmadi M. H. Rudrappa M. (2024). Pleural effusion. Treasure Island (FL): StatPearls Publishing. Available online at: https://www.ncbi.nlm.nih.gov/books/NBK448189/ (Accessed Junuary 22, 2026). 28846252

[B63] Kuivenhoven M. Mason K. (2023). “Arsenic toxicity,” in Treasure Island (FL): StatPearls Publishing. Available online at: https://www.ncbi.nlm.nih.gov/books/NBK541125/ (Accessed February 1, 2026). 31082169

[B64] Lee S. H. Park M. J. Choi S. I. Le E. J. Lee S. Y. In K. H. (2017). Reactive oxygen species modulator 1 (Romo1) as a novel diagnostic marker for lung cancer-related malignant effusion. Med. Baltim. 96 (4), e5975. 10.1097/MD.0000000000005975 28121949 PMC5287973

[B65] Leyssens L. Vinck B. Van Der Straeten C. Wuyts F. Maes L. (2017). Cobalt toxicity in humans-A review of the potential sources and systemic health effects. Toxicology 387, 43–56. 10.1016/j.tox.2017.05.015 28572025

[B66] Li W. Wu J. Zhou Y. Wang Q. Yu F. Wang R. (2023). Chemical characteristics and sources analysis of PM2.5 in shaoxing in winter. Atmosphere 14 (8), 1256. 10.3390/atmos14081256

[B67] Lodovici M. Bigagli E. (2011). Oxidative stress and air pollution exposure. J. Toxicol. 2011, 487074. 10.1155/2011/487074 21860622 PMC3155788

[B68] Lu X. Zhang X. Zhang T. Ni J. Peng Y. Chen X. (2025). Association of short-term exposure to air pollutants with mortality from respiratory diseases: a case-crossover study of individual cases based in Hefei, China. J. Glob. Health 15, 04196. 10.7189/jogh.15.04196 40570205 PMC12201937

[B69] Lyu R. Shi Z. Alam M. S. Wu X. Liu D. Vu T. V. (2019). Insight into the composition of organic compounds (≥C) in PM2.5 in wintertime in Beijing, China. Atmos. Chem. Phys. 19 (16), 10865–10881. 10.5194/acp-19-10865-2019

[B70] Ma N. Wang Y. Li X. Xu M. Tan D. (2025). Reactive oxygen species in cancer: mechanistic insights and therapeutic innovations. Cell Stress Chaperones 30 (5), 100108. 10.1016/j.cstres.2025.100108 40769273 PMC12398932

[B71] Magro G. Laterza V. Tosto F. Torrente A. (2025). Manganese neurotoxicity: a comprehensive review of pathophysiology and inherited and acquired disorders. J. Xenobiot. 15 (2), 54. 10.3390/jox15020054 40278159 PMC12028444

[B72] Martin R. Lee V. R. (2024). “Antimony toxicity,”. Treasure Island (FL): StatPearls Publishing. Available online at: https://www.ncbi.nlm.nih.gov/books/NBK608003/ (Accessed February 10, 2026). 39383292

[B73] Matei E. Râpă M. Mateş I. M. Popescu A. F. Bădiceanu A. Balint A. I. (2025). Heavy metals in particulate matter-trends and impacts on environment. Molecules 30 (7), 1455. 10.3390/molecules30071455 40286077 PMC11990512

[B74] Miserocchi G. (1997). Physiology and pathophysiology of pleural fluid turnover. Eur. Respi.r J. 10 (1), 219–225. 10.1183/09031936.97.10010219 9032518

[B75] Mizuno D. Kawahara M. (2013). The molecular mechanisms of zinc neurotoxicity and the pathogenesis of vascular type senile dementia. Int. J. Mol. Sci. 14 (11), 22067–22081. 10.3390/ijms141122067 24213606 PMC3856052

[B76] Mühlfeld C. Rothen-Rutishauser B. Blank F. Vanhecke D. Ochs M. Gehr P. (2008). Interactions of nanoparticles with pulmonary structures and cellular responses. Am. J. Physiol. Lung Cell Mol. Physiol. 294 (5), L817–L829. 10.1152/ajplung.00442.2007 18263666

[B77] Najeeb Q. Bhaskar N. Masood I. Wadhwa S. Kaur H. Ishaq S. (2012). Malondialdehyde (MDA) superoxide dismutase (SOD) levels - distinguishing parameters between benign malignant pleural effusions. Free Radic. Antioxid. 2 (2), 8–11. 10.5530/ax.2012.2.2.2

[B78] NCBI (2005). Toxicological profile for tin and tin compounds Health effects. Atlanta (GA): Agency for Toxic Substances and Disease Registry US. Available online at: https://www.ncbi.nlm.nih.gov/books/NBK599932/ (Accessed February 10, 2026). 38315787

[B79] NCBI (2019). Toxicological profile for antimony and compounds. Atlanta (GA): Agency for Toxic Substances and Disease Registry US. Available online at: https://www.ncbi.nlm.nih.gov/books/NBK591404/ (Accessed February 10, 2026). 37184169

[B80] NCBI (2020). Toxicological profile for Molybdenum Health Effects. Atlanta (GA): Agency for Toxic Substances and Disease Registry US. Available online at: https://www.ncbi.nlm.nih.gov/books/NBK590366/ (Accessed February 10, 2026). 37040459

[B81] NCBI (2024). Toxicological profile for cobalt. Atlanta (GA): Agency for Toxic Substances and Disease Registry US. Available online at: https://www.ncbi.nlm.nih.gov/books/NBK609991/ (Accessed February 11, 2026). 39652701

[B82] O'Neal S. L. Zheng W. (2015). Manganese toxicity upon overexposure: a decade in review. Curr. Environ. Health Rep. 2 (3), 315–328. 10.1007/s40572-015-0056-x 26231508 PMC4545267

[B83] Olazábal-Morán M. Pérez E. Esteban-Arranz A. Garrido A. (2025). The role of reactive oxygen species in lung cancer development: nanomedicine as a therapeutic strategy. Biomolecules 15 (9), 1316. 10.3390/biom15091316 41008623 PMC12467217

[B84] Papageorgiou E. Kostikas K. Kiropoulos T. Karetsi E. Mpatavanis G. Gourgoulianis K. I. (2005). Increased oxidative stress in exudative pleural effusions: a new marker for the differentiation between exudates and transudates? Chest 128 (5), 3291–3297. 10.1378/chest.128.5.3291 16304274

[B85] Papanikolaou G. Pantopoulos K. (2005). Iron metabolism and toxicity. Toxicol. Appl. Pharmacol. 202 (2), 199–211. 10.1016/j.taap.2004.06.021 15629195

[B86] Pardessus-Otero A. Rafecas-Codern A. Porcel J. M. Serra-Mitjà P. Ferreiro L. Botana-Rial M. (2024). Malignant pleural effusion: a multidisciplinary approach. Open Respir. Arch. 6 (4), 100349. 10.1016/j.opresp.2024.100349 39091982 PMC11293617

[B87] Park J. D. Zheng W. (2012). Human exposure and health effects of inorganic and elemental mercury. J. Prev. Med. Public Health 45 (6), 344–352. 10.3961/jpmph.2012.45.6.344 23230464 PMC3514464

[B88] Pinto E. Cruz M. Ramos P. Santos A. Almeida A. (2017). Metals transfer from tobacco to cigarette smoke: evidences in smokers' lung tissue. J. Hazard. Mater. 325, 31–35. 10.1016/j.jhazmat.2016.11.069 27914289

[B89] Pizzino G. Irrera N. Cucinotta M. Pallio G. Mannino F. Arcoraci V. (2017). Oxidative stress: harms and benefits for human health. Oxid. Med. Cell Longev. 2017, 8416763. 10.1155/2017/8416763 28819546 PMC5551541

[B90] Plum L. M. Rink L. Haase H. (2010). The essential toxin: impact of zinc on human health. Int. J. Environ. Res. Public Health 7 (4), 1342–1365. 10.3390/ijerph7041342 20617034 PMC2872358

[B91] Poole L. B. (2015). The basics of thiols and cysteines in redox biology and chemistry. Free. Radic. Biol. Med. 80, 148–157. 10.1016/j.freeradbiomed.2014.11.013 25433365 PMC4355186

[B92] Porcel J. M. Light R. W. (2006). Light RW. Diagnostic approach to pleural effusion in adults. Am. Fam. Physician 73 (7), 1211–1220. 10.1378/chest.105.5.1338 16623208

[B93] Rahman I. Biswas S. K. Kode A. (2006). Oxidant and antioxidant balance in the airways and airway diseases. Eur. J. Pharmacol. 533 (1-3), 222–239. 10.1016/j.ejphar.2005.12.087 16500642

[B94] Reddy V. N. Kumar V. A. Srinivas M. Reddy V. N. (2009). Study on oxidative metabolic changes to differentiate exudative from transudative pleural effusions. S. J. Pharm. Sci. 1 (1), 38–43. 10.3329/sjps.v1i1.1806

[B95] Renke G. Almeida V. B. P. Souza E. A. Lessa S. Teixeira R. L. Rocha L. (2023). Clinical outcomes of the deleterious effects of aluminum on Neuro-Cognition, inflammation, and health: a review. Nutrients 15 (9), 2221. 10.3390/nu15092221 37432384 PMC10180736

[B96] Royer A. Sharman T. (2023). “Copper toxicity,” in StatPearls (Treasure Island (FL): StatPearls Publishing). Available online at: https://www.ncbi.nlm.nih.gov/books/NBK557456/ (Accessed February 14, 2026). 32491388

[B97] Sakrajda K. Rybakowski J. K. (2025). The mechanisms of lithium action: the old and new findings. Pharm. (Basel) 18 (4), 467. 10.3390/ph18040467 PMC12030015 40283904

[B98] Sasso A. F. Isukapalli S. S. Georgopoulos P. G. (2010). A generalized physiologically-based toxicokinetic modeling system for chemical mixtures containing metals. Theor. Biol. Med. Model 7, 17. 10.1186/1742-4682-7-17 20525215 PMC2903511

[B99] Şener M. U. Sönmez Ö. Keyf İ. A. Erel Ö. Alışık M. Bulut S. (2020). Evaluation of Thiol/Disulfide homeostasis in lung cancer. Turk. Thorac. J. 21 (4), 255–260. 10.5152/TurkThoracJ.2019.19033 32687786 PMC7371402

[B100] Shin D. Y. Lee S. M. Jang Y. Lee J. Lee C. M. Cho E. M. (2023). Adverse human health effects of chromium by exposure route: a comprehensive review based on toxicogenomic approach. Int. J. Mol. Sci. 24 (4), 3410. 10.3390/ijms24043410 36834821 PMC9963995

[B101] Sies H. Berndt C. Jones D. P. (2017). Oxidative stress. Annu. Rev. Biochem. 86, 715–748. 10.1146/annurev-biochem-061516-045037 28441057

[B102] Solari E. Marcozzi C. Ottaviani C. Negrini D. Moriondo A. (2022). Draining the pleural space: lymphatic vessels facing the Most challenging task. Biol. (Basel). 11 (3), 419. 10.3390/biology11030419 PMC8945018 35336793

[B103] Steck M. B. Murray B. P. (2024). “Silver toxicity,” in StatPearls (Treasure Island (FL): StatPearls Publishing). Available online at: https://www.ncbi.nlm.nih.gov/books/NBK604211/ (Accessed February 14, 2026). 38861631

[B104] Tekşen F. Mungan D. Sayal A. Misurligil Z. Aydin A. Gürbüz L. (1996). Serum and pleural fluid selenium, copper, zinc, and magnesium levels in malignant and nonmalignant pleural diseases. Respiration 63 (1), 25–27. 10.1159/000196511 8833989

[B105] Thandra K. C. Barsouk A. Saginala K. Aluru J. S. Barsouk A. (2021). Epidemiology of lung cancer. Contemp. Oncol. Pozn. 25 (1), 45–52. 10.5114/wo.2021.103829 33911981 PMC8063897

[B106] Toxicological Profile for Vanadium (2012). Health effects. Atlanta (GA): Agency for Toxic Substances and Disease Registry US. Available online at: https://www.ncbi.nlm.nih.gov/books/NBK592340/ (Accessed February 15, 2026). 37262203

[B107] Vali R. Shirvanian K. Farkhondeh T. Aschner M. Samini F. Samarghandian S. (2025). A review study on the effect of zinc on oxidative stress-related neurological disorders. J. Trace Elem. Med. Biol. 88, 127618. 10.1016/j.jtemb.2025.127618 39978164

[B108] Wang M. Zhang Z. Wang X. (2014). Superoxide dismutase 2 as a marker to differentiate tuberculous pleural effusions from malignant pleural effusions. Clin. (Sao Paulo) 69 (12), 799–803. 10.6061/clinics/2014(12)02 25627990 PMC4286673

[B109] Wang Y. Qi H. Liu Y. Duan C. Liu X. Xia T. (2021). The double-edged roles of ROS in cancer prevention and therapy. Theranostics 11 (10), 4839–4857. 10.7150/thno.56747 33754031 PMC7978298

[B110] World Health Organization (2018). World health organization. Available online at: https://www.who.int/news-room/fact-sheets/detail/ambient-(outdoor)-air-quality-and-health (Accessed January 5, 2026).

[B111] Wu Z. Liu D. Zhao T. Su Y. Zhou B. (2021). Size distributions of water-soluble inorganic ions in atmospheric aerosols during the Meiyu period in the Yangtze River Delta, China. Front. Environ. Sci. 9, 788115. 10.3389/fenvs.2021.788115

[B112] Xu J. Wise J. T. F. Wang L. Schumann K. Zhang Z. Shi X. (2017). Dual roles of oxidative stress in metal carcinogenesis. J. Environ. Pathol. Toxicol. Oncol. 36 (4), 345–376. 10.1615/JEnvironPatholToxicolOncol.2017025229 29431065 PMC6361383

[B113] Yadav M. M. (2019). Comparative study of transudate and exudate pleural fluid using C-reactive protein, uric acid and Malondialdehyde as markers. Indian J. Med. Biochem. 23 (1), 193–196. 10.5005/jp-journals-10054-0084

[B114] Yang Y. Hassan M. F. Ali W. Zou H. Liu Z. Ma Y. (2025). Effects of cadmium pollution on human health: a narrative review. Atmosphere 16 (2), 225. 10.3390/atmos16020225

[B115] Yokel R. A. Lasley S. M. Dorman D. C. (2006). The speciation of metals in mammals influences their toxicokinetics and toxicodynamics and therefore human health risk assessment. J. Toxicol. Environ. Health B. Crit. Rev. 9 (1), 63–85. 10.1080/15287390500196230 16393870

[B116] Yordanov A. Tsoneva E. (2025). ROMO1: a distinct mitochondrial protein with dual roles in dynamics and function. Antioxidants (Basel) 14 (5), 540. 10.3390/antiox14050540 40427422 PMC12108320

[B117] Zalewska-Ziob M. Adamek B. Kasperczyk J. Romuk E. Hudziec E. Chwalińska E. (2019). Activity of antioxidant enzymes in the tumor and adjacent noncancerous tissues of non-small-cell lung cancer. Oxid. Med. Cell Longev. 2019, 2901840. 10.1155/2019/2901840 31781331 PMC6875225

[B118] Zambelli B. Uversky V. N. Ciurli S. (2016). Nickel impact on human health: an intrinsic disorder perspective. Biochim. Biophys. Acta. 1864 (12), 1714–1731. 10.1016/j.bbapap.2016.09.008 27645710

[B119] Zhang X. Wang S. Ling L. Hou G. Leng S. Ma N. (2022). The distribution and structural fingerprints of metals from particulate matters (PM) deposited in human lungs. Ecotoxicol. Environ. Saf. 233, 113324. 10.1016/j.ecoenv.2022.113324 35193030

[B120] Zhang F. Guo C. G. Yang C. Wang F. Wang W. Zhang L. (2025). Exposure-response associations of ambient heavy metal and persistent organic pollutant with all-cause and cause-specific mortality: a prospective cohort study. Environ. Health (Wash). 3 (5), 493–503. 10.1021/envhealth.4c00191 40400556 PMC12090017

[B121] Zhang F. Wang J. Fu J. Hu L. Zheng X. Wang Y. (2020). Clinical value of combined detection of reactive oxygen species modulator 1 and adenosine deaminase in pleural effusion in the identification of NSCLC associated malignant pleural effusion. J. Clin. Lab. Anal. 34 (3), e23091. 10.1002/jcla.23091 31709646 PMC7083413

[B122] Zhang Z. Zhu D. Cui B. Ding R. Shi X. He P. (2020). Association between particulate matter air pollution and lung cancer. Thorax 75 (1), 85–87. 10.1136/thoraxjnl-2019-213722 31727788

